# Putative C_2_H_2_ Transcription Factor *AflZKS3* Regulates Aflatoxin and Pathogenicity in *Aspergillus flavus*

**DOI:** 10.3390/toxins14120883

**Published:** 2022-12-17

**Authors:** Liuke Liang, Haojie Yang, Shan Wei, Shuaibing Zhang, Liang Chen, Yuansen Hu, Yangyong Lv

**Affiliations:** College of Biological Engineering, Henan University of Technology, Zhengzhou 450001, China

**Keywords:** *Aspergillus flavus*, C_2_H_2_ transcription factor, *AflZKS3*, aflatoxin, pathogenicity

## Abstract

Aflatoxin is a carcinogenic secondary metabolite that poses a serious threat to human and animal health. Some C_2_H_2_ transcription factors are associated with fungal growth and secondary metabolic regulation. In this study, we characterized the role of *AflZKS3*, a putative C_2_H_2_ transcription factor based on genome annotation, in the growth and aflatoxin biosynthesis of *A. flavus* and explored its possible mechanisms of action. Surprisingly, the protein was found to be located in the cytoplasm, and gene deletion in *A. flavus* resulted in defective growth and conidia formation, as well as increased sensitivity to the fluorescent brightener Calcofluor white, Congo red, NaCl, and sorbitol stress. Notably, the biosynthesis of aflatoxin B_1_ was completely inhibited in the Δ*AflZKS3* deletion strain, and its ability to infect peanut and corn seeds was also reduced. RNA sequencing showed that differentially expressed genes in the Δ*AflZKS3* strain compared with the control and complementation strains were mainly associated with growth, aflatoxin biosynthesis, and oxidative stress. Thus, *AflZKS3* likely contributes to growth, cell development, and aflatoxin synthesis in *A. flavus*. These findings lay the foundation for a deeper understanding of the roles of C_2_H_2_ transcription factors in *A. flavus* and provide a potential biocontrol target for preventing aflatoxin contamination.

## 1. Introduction

A characteristic of fungi is the ability to produce a wide variety of secondary metabolites, including beneficial compounds such as lovastatin, as well as toxic molecules such as mycotoxins [1]. *Aspergillus flavus*, a conditional fungal pathogen of important crops in pre- and post-harvest periods, produces carcinogenic aflatoxins (AFs) that cause severe yield reduction and represent a serious threat to animal and human health [2]. A study by the Food and Agriculture Organization proved that about a quarter of the world’s total food production is contaminated by mycotoxins each year, and the main source of pollution is *A. flavus* and its secondary metabolites [3]. Therefore, exploring the complex mechanism and regulatory network of AF biosynthesis will help to develop effective measures to control the growth of *A. flavus* and AF contamination, protecting human and animal health and reducing huge economic losses to agricultural production.

The biosynthesis of AFs is regulated by global and pathway-specific transcription factors. Pathway-specific transcription factors, including *aflR* and *aflS* within the AF gene cluster, have been studied extensively [4]. AflR is a DNA-binding zinc cluster protein that binds to a palindromic sequence in the promoter region to activate gene expression [5,6]. AflR is necessary for AF synthesis, and the deletion of *aflR* leads to the downregulation of genes and the complete loss of AF synthesis [7]. AflS regulates genes in the AF synthesis gene cluster by assisting the localization of *aflR* [8]. Additionally, the biosynthesis of AFs is also regulated by global transcription factors such as zinc finger, bZIP, PHD, homeobox, and APSES transcription factors [9,10,11,12]. Among them, the zinc finger family is the largest and includes the Cys_2_His_2_ (C_2_H_2_), Cys_4_ (C_4_), and Zn(Ⅱ)_2_C_6_ subfamilies [13]. Researchers have identified some zinc finger transcription factors with global regulatory functions. The transcription factors nsdC and nsdD, essential for the development of *A. nidulans*, are also involved in the growth and development of *A. flavus*, as well as secondary metabolism. AF production is completely lost in *nsdC*-deleted strains, and *aflD*, *aflM*, and *aflP* genes are not expressed [14]. *MtfA* encodes a C_2_H_2_ zinc finger transcription factor that influences the production of sterigmatocystin, and the overexpression of *mtfA* can dramatically decrease secondary metabolites such as AFB_1_ [1]. RsrA, a highly conserved C_2_H_2_ transcription factor in *A. nidulans*, regulates the synthesis of sterigmatocystin, a precursor of AF [15]. These results suggest that C_2_H_2_ transcription factors play regulatory roles in mycelia growth development and secondary metabolism. Genome annotation (http://ftfd.snu.ac.kr/index.php?a=view, accessed on 10 October 2022) revealed a putative C_2_H_2_ zinc finger transcription factor encoded by *AflZKS3* in the genome of *A. flavus*, which shares 83% homology with the IFM54703_5628 gene in *A. lentulus* with the property of zinc finger protein with KRAB and SCAN domains 3 (http://FungiDB.org, accessed on 10 October 2022); however, its potential functions in growth and AF biosynthesis remain poorly understood.

In this study, the putative C_2_H_2_ zinc finger transcription factor encoded by *AflZKS3* in *A. flavus* was characterized, and its intracellular localization and roles in pathogenicity were investigated. Compared with control and complementation strains, *AflZKS3* deletion strains showed a reduced growth rate and conidia number, an inability to produce AF, and increased sensitivity to Calcofluor white (CFW) and NaCl stress. The pathogenicity of the deletion mutant was decreased when infecting peanuts and maize. RNA sequencing (RNA-seq) transcriptomic analysis showed that differentially expressed genes (DEGs) in the *AflZKS3* deletion strain were mainly associated with growth, oxidative stress, and the biosynthesis of secondary metabolites, including AF and gliotoxin. Our results reveal the potential regulatory mechanism of *AflZKS3* in *A. flavus* growth, cell development, and AF biosynthesis and provide a potential target for controlling *A. flavus* and AF contamination.

## 2. Results

### 2.1. Identification of Putative C_2_H_2_ Zinc Finger Transcription Factor AflZKS3 in A. flavus

**Figure 1:**
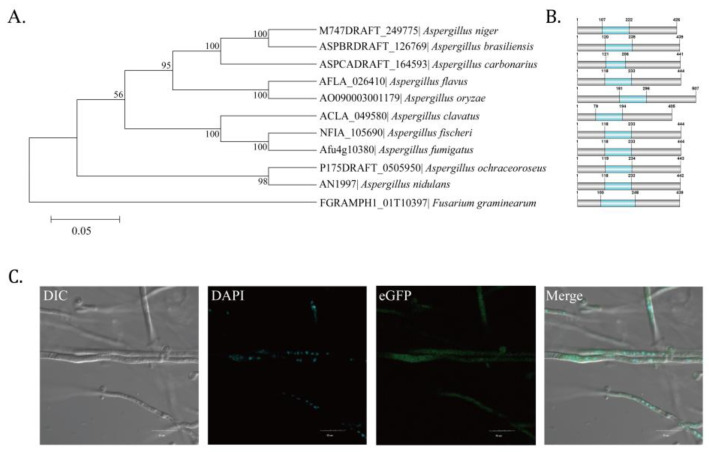
Bioinformatics analyses and subcellular localization of *AflZKS3*. (**A**) Construction of phylogenetic trees of *AflZKS3*. (**B**) Functional domain of *AflZKS3*. The blue area represents the C_2_H_2_ finger domain. (**C**) Localization of *AflZKS3*-eGFP in *A. flavus*.

Homologous genes of *A. flavus* *AflZKS3* were obtained from NCBI by a BLAST search, and sequences were used to construct a phylogenetic tree using the MEGA 6.0 software, which showed that *AflZKS3* was most closely related to *AflZKS3* of *A. oryzae* AO090003001179 (Figure 1A). Protein domain analysis showed that homologs in 10 species harbor C_2_H_2_ finger domains (Figure 1B). Unexpectedly, subcellular localization results demonstrated that *AflZKS3* was not localized in the nucleus, even though it contains a conserved C_2_H_2_ finger domain (Figure 1C).

### 2.2. Deletion of AflZKS3 Affects Growth, Production of Conidia, and AF Biosynthesis

**Figure 2:**
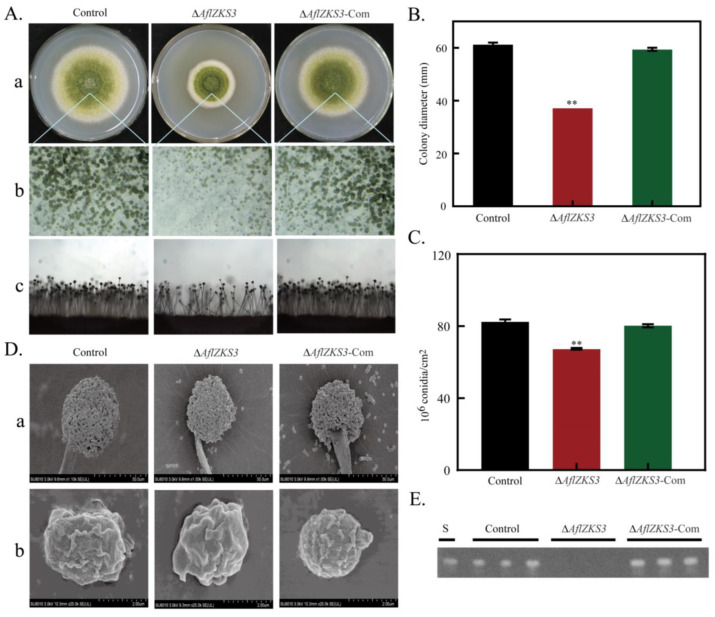
AFLA 026410 affects growth, as well as the conidial and AF biosynthesis of *A. flavus*. (**A**) The colonies: (**a**) stereoscopic microscope, (**b**) conidiophores, and (**c**) analysis of the *A. flavus* control, Δ*AflZKS3*, and Δ*AflZKS3*-Com strains. (**B**) Colony diameter. (**C**) Conidial production. (**D**) SEM analysis of (**a**) conidia and (**b**) conidial heads. (**E**) TLC analysis of AFB_1_ production. ** represents *p* < 0.001.

In order to study the roles of *AflZKS3* in the pathogenicity of *A. flavus*, we constructed deletion and complementation strains and verified them using PCR (Figure S1). The role of *AflZKS3* in the growth, development, and conidia formation of *A. flavus* was further studied, and *A. flavus* control, Δ*AflZKS3*, and Δ*AflZKS3*-Com spore suspensions were inoculated and inverted for 5 days at 30 °C. The results demonstrated that compared with the *A. flavus* control and Δ*AflZKS3*-Com strains, the mycelia of the Δ*AflZKS3* strain were tight, the edges were regular, and the colony diameter was significantly reduced, which indicates that *AflZKS3* plays a significant inhibitory role in the growth of *A. flavus*. Spore analysis indicated that the lack of the *AflZKS3* gene reduced the sporogenic ability of *A. flavus*, consistent with the results observed by stereoscopic microscopy (Figure 2A–C). Additionally, SEM images showed that deletion of the *AflZKS3* gene had a minor effect on the morphology of conidia and apical spore heads (Figure 2D). TLC analysis showed that the Δ*AflZKS3* strain did not emit fluorescence, indicating that *AflZKS3* is essential for AF production in *A. flavus* (Figure 2E).

### 2.3. The ΔAflZKS3 Deletion Mutant Is Highly Sensitive to CFW and NaCl

**Figure 3:**
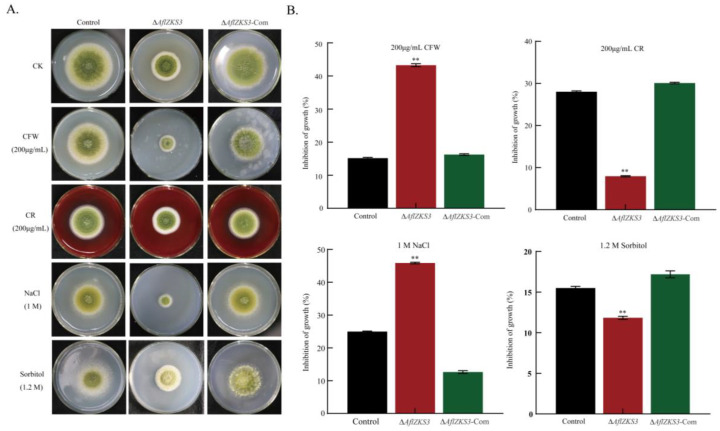
Comparison of multiple stress sensitivity of *A. flavus* control, Δ*AflZKS3*, and Δ*AflZKS3*-Com strains. (**A**) The growth of *A. flavus* strains on PDA media supplemented with CK, CFW, CR, NaCl, and sorbitol, respectively. (**B**) Colony diameter. ** represents *p* < 0.001.

To investigate the effects of *AflZKS3* on the cell wall of *A. flavus*, strains were cultured in PDA medium supplemented with the cell-wall-stress reagents CFW and CR for 5 days. The results showed that the *AflZKS3* deletion strain was sensitive to CFW compared with the control strain and the *AflZKS3*-Com strain. In addition, similar changes were also observed in the *AflZKS3* deletion strain in response to NaCl stress. Specifically, the Δ*AflZKS3* strain was much more sensitive to NaCl, and the colony diameter was significantly reduced compared with CR and sorbitol treatment. However, the ∆*AflZKS3* mutant showed less sensitivity to CR and sorbitol treatment than the control strain and the *AflZKS3*-Com strain (Figure 3). These results show that *AflZKS3* may have a function in maintaining the cell wall integrity of *A. flavus*.

### 2.4. Effects of AflZKS3 Deletion on the Pathogenicity of A. flavus on Grain Seeds

**Figure 4:**
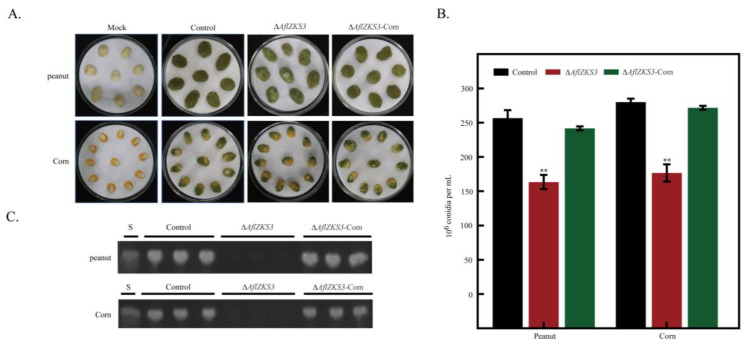
Effect of *AflZKS3* deletion on the ability of *A. flavus* to infect peanut and corn seeds. (**A**) Colonization of *A. flavus* control, Δ*AflZKS3*, and Δ*AflZKS3*-Com strains on peanut and corn seeds. (**B**) Conidia number. (**C**) TLC analysis of AFB_1_ production. ** represents *p* < 0.001.

To study the function of *AflZKS3* in the growth and AFB_1_ biosynthesis of *A. flavus* in peanut and corn seeds, spore suspensions were inoculated, and AF biosynthesis was characterized. The results showed that the surface of peanuts and corn infected with *A. flavus* control and Δ*AflZKS3*-Com strains produced a large number of tight green spores. The surface spores of peanuts and corn infected with the *A. flavus* Δ*AflZKS3* strain were looser, and the yield of conidia decreased by 30.43% and 31.33%, respectively (Figure 4A,B). Additionally, TLC analysis indicated that the deletion of *AflZKS3* totally blocked the biosynthesis of AFB_1_ (Figure 4C). These results suggest that *AflZKS3* affected *A. flavus* pathogenicity by inhibiting *A. flavus* colonization and AF production.

### 2.5. Transcriptome Analysis

Transcriptome analysis was conducted to investigate the underlying mechanism of *AflZKS3* deletion on the growth and AF biosynthesis in *A. flavus*. The Pearson correlation coefficient was greater than 0.825 between any two replicates, indicating that expression patterns were similar among samples in the groups, and biological replicates were qualified for subsequent analysis (Figure S2A). Expression levels of genes were normalized by FPKM, and DEGs were compared (Figure S2B). The volcano map in Figure S2 shows gene expression fold changes and significance. A total of 1326 significant DEGs were identified, including 476 upregulated genes (35.90%) and 850 downregulated genes (64.10%) (Figure S2D).

**Figure 5:**
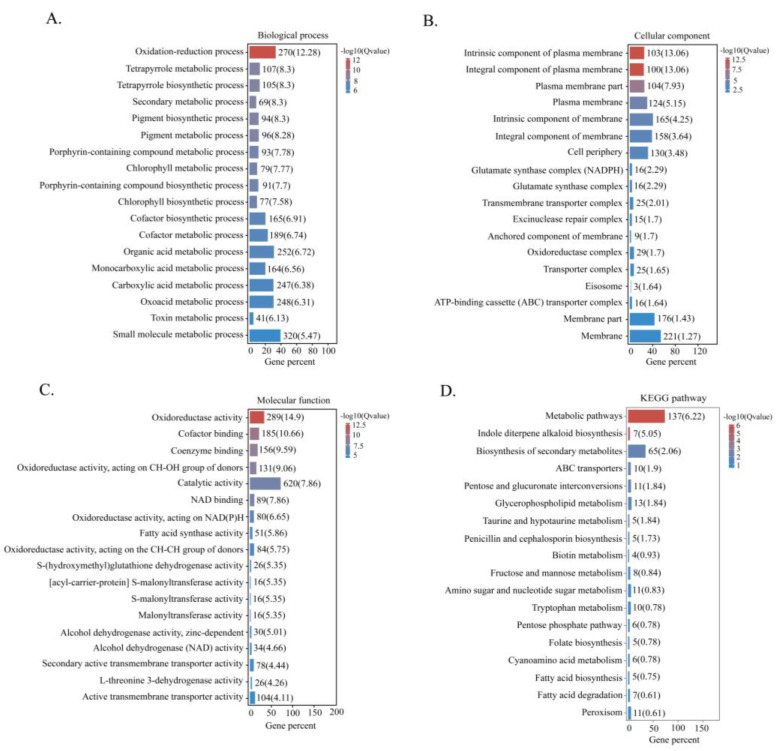
Functional enrichment analysis of DEGs. (**A**) Biological process. (**B**) Cellular component. (**C**) Molecular function. (**D**) KEGG pathway.

GO functional enrichment analysis was performed to further investigate the biological functions of the DEGs. The results revealed that DEGs were mainly associated with oxidation–reduction and metabolic biological processes (Figure 5A). The most enriched cell component categories were the plasma membrane and the cell periphery and membrane (Figure 5B). Molecular functions were mainly related to oxidoreductase activity, catalytic activity, and binding (Figure 5C). Additionally, KEGG pathway enrichment analysis showed that DEGs were mainly linked to metabolic pathways and the biosynthesis of secondary metabolites (Figure 5D).

### 2.6. Categorisation of DEGs

In order to further unveil the regulatory mechanisms of *AflZKS3* in growth and AF biosynthesis, representative DEGs were categorized into four groups: growth, cell wall, secondary metabolism, and oxidative stress (Table 1).

Genes influenced by C_2_H_2_ participated in growth and development. The results indicate that the growth-related genes, *FLOT1*, *freB*, *aspC*, and *psd2*; conidia formation genes, *vosA*, *con-6*, *cetA*, *DIT2*, *AQY1*, and *betA*; and the regulated conidia lipid homeostasis gene, *SAY1*, were downregulated. Additionally, *chiA*, *agn1*, *gel2*, *gel4*, *glx3*, and *gpi13*, involved in fungal cell wall formation and integrity, were also downregulated.

Alongside defective growth, AF biosynthesis was altered. The results demonstrated that the AFB_1_ biosynthetic pathway genes, *fasA*, *aflQ*, *aflB*, and *aflF*, as well as the genes encoding O-methyltransferase (*imqG*, *aclH*) and cytochrome P450 (*lnaC*, *BOT4*), were downregulated in the *AflZKS3* deletion strain. The biosynthesis of other secondary metabolites was also affected; gliotoxin (*gliA*), polyketide (*albA*, *nscA*, and *pksCT*), and non-ribosomal polypeptide (*lnaA)* genes were downregulated. Additionally, the deletion of *AflZKS3* also downregulated antioxidant-related genes (*sodB*, *cat1*, *oxr1*, and *ssuD*) and salt stress-related genes (*dur3*, *phoD*).

### 2.7. Validation of RNA-Seq

**Figure 6:**
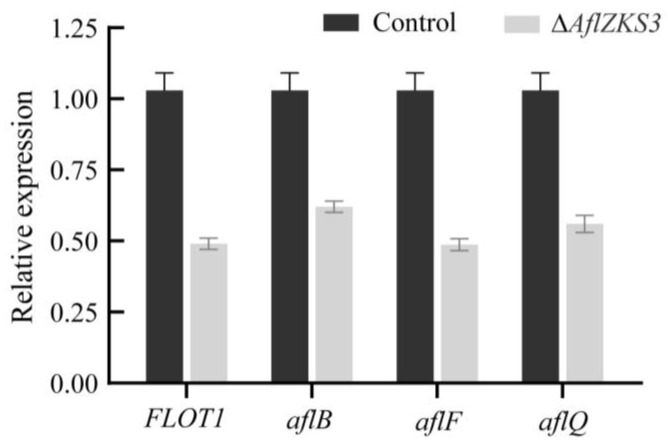
qRT-PCR analysis to validate four genes with RNA-seq data (growth-related gene *FLOT1*, and AF biosynthesis-related genes *aflB*, *aflF*, and *aflQ*).

To further verify the expression levels of the DEGs identified in the transcriptomic analysis, one growth-related gene and three AF synthesis pathway genes were selected, and their expression levels were verified by qRT-PCR. The results showed that the qRT-PCR results were consistent with the transcriptomic results, and the expressions of selected genes were downregulated (Figure 6).

## 3. Discussion

C_2_H_2_ zinc finger transcription factors are known to play vital roles in the development and pathogenicity of microorganisms [16]. In this study, the putative C_2_H_2_ zinc finger transcription factor, *AflZKS3*, annotated in the *A. flavus* genome, was characterized. The results indicated that this transcription factor is not located in the nucleus and that it plays a major role in the growth and cell development of *A. flavus* and in AF biosynthesis. Additionally, the potential mechanism was explored by RNA-seq analysis.

*AflZKS3* was annotated as a putative C_2_H_2_ zinc finger transcription factor in the *A. flavus* genome. The sequence alignment of homologous *Aspergillus*, *Fusarium*, and *Saccharomyces* proteins revealed that *AflZKS3* possesses a conserved C_2_H_2_ finger domain, implying similar functions. In *S. cerevisiae* [17] and *A. nidulans* [18], C_2_H_2_ transcription factors are located in the nucleus, but unexpectedly, our results revealed that *AflZKS3* was not located in the nucleus. Previous studies have shown that the localization pattern of the iron deficiency-induced transcription factor, bHLH039, in *Arabidopsis* varies according to the presence of Fer-like iron deficiency-induced transcription factor (FIT) and that bHLH039 is primarily localized in the cytoplasm when expressed in cells lacking FIT, but localized in the nucleus when FIT is present [19]. These results suggested that the subcellular localization of *AflZKS3* might be influenced by other regulatory factors resembling bHLH039 in *Arabidopsis*. We further determined subcellular localization in the presence of CFW, NaCl, and sorbitol and found that *AflZKS3* was not located in the nucleus (Figure S3). However, the specific reasons remain to be further explored.

C_2_H_2_ transcription factors have been shown to play crucial roles in plant and fungal growth and development [20]. Previous studies have reported that the membrane microdomain-associated protein Flotillin 1 (Flot1) is involved in plant growth and development in *A. thaliana* [21]. In *A. fumigatus*, the *freB* gene encoding iron reductase mediates iron metabolism, and the disruption of *freB* reduces the fungal growth rate, iron reductase activity, and tolerance to oxidative stress [22]. Septins are a conserved GTPase family that play vital roles in growth, meristem, and cell wall integrity. In *A. fumigatus*, the loss of *aspC* led to septation, cell wall stress, and meristem defects [23]. Phosphatidylserine decarboxylases (PSDs) are responsible for catalyzing the production of phosphatidylethanolamine, an important phospholipid in homeostasis, growth, and the development of fungi. In *A. nidulans*, the loss of *psdB* resulted in severe growth defects, impaired conidia development, and abnormal conidia structure [24]. The present study found that growth-related genes such as *FLOT1*, *freB*, *aspC*, and *psd2* were downregulated after the deletion of *AflZKS3*, indicating that iron metabolism, GTPases, and phospholipid homeostasis might be regulated by *AflZKS3*, and thereby affect mycelia growth. Additionally, *con-6*, a conidia-related gene, is relatively conserved in filamentous fungi and preferentially expressed during conidia development [25]. The *vosA* gene encodes a key regulator of *Aspergillus* spores and is essential for the morphological development and metabolic integrity of conidia. Previous studies found that a *vosA* mutant strain displayed defective growth on media supplemented with Congo red, sodium chloride, and sorbitol [26]. Herein, we found that genes associated with spore development in the strain Δ*AflZKS3*, such as *con-6*, *vosA*, *cetA*, *betA*, *AQY1*, spore wall-specific gene *DIT2* [27], and conidia lipid homeostasis-related gene *SAY1* [28] were all downregulated. Furthermore, previous studies indicated that CFW is specifically bound to chitin, while CR is bound to β-1, 3-glucan, thus obstructing the normal assembly of the cell wall, resulting in cell wall stress, and inhibiting the growth of the cell [29]. Our results demonstrated that the *AflZKS3* deletion strain showed different sensitivity to CFW and CR compared with *A. flavus* control and the *AflZKS3*-Com strains, which might be attributed to their different mechanisms of action and the cell wall defects caused by *AflZKS3* deletion. Additionally, the *AflZKS3* deletion strain was much more sensitive to NaCl than sorbitol compared with the control and *AflZKS3*-Com strains. The possible reason might be that NaCl belongs to the category of ionic and cell penetrating agent, which can induce ionic stress and produce specific ionic toxicity, while sorbitol belongs to non-ionic osmotic stress agent. Previous research also demonstrated that the induced effect of NaCl is more profound than that of sorbitol in *Japonica* rice [30,31].

The cell wall of fungi is a complex structure composed mainly of chitin and glucan and plays a vital role in morphogenesis and protection from various environmental stresses [32]. *ChiA* is a class III chitinase involved in spore germination and mycelial growth [33]. *Agn1*, which encodes 1, 3-α-glucanase, is involved in cell division [34]. *Gel2* and *glx3* are associated with cell wall integrity [35,36]. These results indicate that *AflZKS3* might affect fungal morphogenesis, defense responses, and cell division by downregulating cell wall-related genes *chiA*, *agn1*, *gel2*, *gel4*, *glx3*, and *gpi13*. We found that the *AFLA_02641* deletion mutant displayed increased sensitivity to CFW, similar to the *glx3* deletion strain in *Candida albicans* [36].

*A. flavus* growth has been reported to be closely related to AF biosynthesis [37]. Impaired growth and conidia development are often accompanied by secondary metabolism disruption. The biosynthesis of AFs is a complex enzymatic process involving 21 enzymes encoded by a gene cluster ~70 kb in size [38]. Studies have shown that the biosynthesis of fatty acids is involved in the initial stage of biosynthesis, fatty acid synthase is involved in the formation of polyketide initiation units of AFs, and high fatty acid synthase activity can promote AFB_1_ production [39]. Furthermore, *fas-1*, which encodes fatty acid synthase, is required for the biosynthesis of norsolorinic acid and AFs [40]. *AflQ* encodes an oxidoreductase involved in the formation of the AFB_1_ precursor hydroxyl-methylsterigmatocystin, and it plays a role in the latter stages of the biosynthetic pathway [41]. There is a strong linear relationship between *aflQ* expression and the AF-producing capacity of *A. flavus* and *A. parasiticus* [42]. In this study, the deletion of *AflZKS3* downregulated the AF biosynthesis-related genes, *fasA*, *aflQ*, *aflB*, and *aflF*. O-methyltransferase, another key enzyme in AFB_1_ synthesis, catalyzes the transformation of sterigmatocystin to O-methylsterigmatocystin and dihydrosterigmatocystin to dihydro-O-methylsterigmatocystin [38]. Cytochrome P450 enzymes are involved in the formation of sterigmatocystin, a late intermediate in the AFB_1_ synthesis pathway [43]. We found that genes associated with O-methyltransferase (*imqG*, *aclH*) and cytochrome P450 (*lnaC*, *BOT4*) were downregulated in *AflZKS3* mutants. These results indicate that AFB_1_ production can be inhibited by *AflZKS3* through the regulation of multiple genes involved in AF biosynthesis.

In addition to AF biosynthesis, genes involved in other secondary metabolic pathways were also affected. Gliotoxin is synthesized by a biosynthetic gene cluster of 12 genes in *A. fumigatus* [44]. *GliA* is involved in gliotoxin biosynthesis and has important functions in gliotoxin export and fungal self-protection. It was found that the disruption of *gliA* greatly reduced the production of gliotoxin [45]. We found that the *gliA* gene related to gliotoxin biosynthesis was downregulated. Additionally, polyketide synthases and non-ribosomal peptide synthetases are large multimodular enzymes that participate in the biosynthesis of polyketides and peptide secondary metabolites [46]. Among them, polyketides are the most abundant fungal secondary metabolites, and they are synthesized by a type I diketone synthase [43]. Previous studies have revealed that the deletion of the *pksCT* gene in *Monascus* decreased citrinin production capacity by >98% [47]. In this study, genes associated with polyketide synthase and non-ribosomal peptide synthase (*albA*, *nscA*, *pksCT*, and *lnaA*) were also downregulated. These results suggest that *AflZKS3* might play a global regulatory role in mycotoxin export and the self-protection of fungi.

Oxidative stress in filamentous fungi is often associated with secondary metabolism, and it is also one of the prerequisites for AF production. Studies have found that low concentrations of reactive oxygen species (ROS) can stimulate the synthesis of secondary metabolites; conversely, high concentrations of ROS are toxic to cells, even causing cell death, and they are detrimental to the biosynthesis of secondary metabolites [48]. A variety of antioxidant enzymes produced by cells, such as superoxide dismutase, peroxidase, and catalase, remove excess ROS to protect cells from oxidative stress [49]. Previous studies have indicated that *oxr1* encodes an antioxidant regulator that protects against intracellular H_2_O_2_-induced oxidative stress [50]. *SsuD* encodes an alkane sulfonate monooxygenase that also protects cells from oxidative stress [51]. In our current study, transcriptome data showed that the deletion of *AflZKS3* downregulated antioxidant-related genes *sodB*, *cat1*, *oxr1*, and *ssuD*, and salt stress-related genes *dur3* and *phoD* [52,53], which might be responsible for the observed changes in AFB_1_ and other secondary metabolites.

In conclusion, we investigated the putative C_2_H_2_ zinc finger transcription factor *AflZKS3* in *A. flavus*, and the results indicated that deletion of *AflZKS3* inhibited cell growth, conidia formation, and AFB_1_ biosynthesis ability. RNA-seq was used to further investigate its underlying regulatory mechanism, and the analysis of DEGs indicated that growth-related genes (*FLOT1*, *psd2*, *vosA*, *con-6,* and *gel2*), secondary metabolism-related genes (*aflB*, *aflF*, *aflQ*, and *pksCT*), and oxygen stress-related genes (*sodB*, *cat1*, and *oxr1*) were downregulated. Therefore, the putative C_2_H_2_ zinc finger transcription factor *AflZKS3* regulates growth, cell development, and oxidative stress-related genes, and affects the secondary metabolism in *A. flavus*. These results further our understanding of the functions of C_2_H_2_ zinc finger transcription factors in fungal pathogenicity regulation and provide a potential target for developing novel control strategies in *A. flavus*.

## 4. Materials and Methods

### 4.1. Strains, Media, and Culture Conditions

*A. flavus* strain CA14 (*kusA^−^, pyrG^+^*) served as the control strain, and *AflZKS3* deletion (Δ*AflZKS3*), *AflZKS3* complementation (Δ*AflZKS3*-Com), and *AflZKS3*-eGFP strains were explored in this study. Potato dextrose agar (PDA) was used to evaluate growth rate, conidia number, and AFB_1_ yield, with a final concentration of 10 mM uridine added if necessary. CFW, NaCl, and sorbitol were added to PDA medium to assess sensitivity to stress. *A. flavus* transformation was carried out according to previous methods [54]. All experiments were independently repeated three times.

### 4.2. Sequence Homology Analysis

The sequence of *AflZKS3* was searched against the NCBI database, and BLAST comparison was performed to obtain homologous sequences. Relationships were analyzed using MEGA 6.0. software (Mega Limited, Auckland, New Zealand). Protein-related information was downloaded to explore *AflZKS3* domains, and a protein domain comparison map was generated using DOG 2.0 software (University of Science & Technology of China, Anhui, China).

### 4.3. Construction of Deletion, Complementation, and Localization Strains

Deletion, complementation, and localization strains were constructed using the primers listed in Table S1. For the deletion strain, the *pyrG* gene was used to replace the *AflZKS3* target gene in the *A. flavus* genome. Primers *AflZKS3*-del-1 and *AflZKS3*-del-2 were used to amplify the 1424 bp upstream flanking region of the target gene, and primers *AflZKS3*-del-3 and *AflZKS3*-del-4 were used to amplify the 1423 bp downstream flanking region. *PyrG* screening marker genes were amplified from plasmid ANIp7 with primers pyrG-F and pyrG-R. The flanking regions, *pyrG* screening marker gene, and downstream homologous arm were ligated according to a previous study [54], and products were purified and transferred into the *A. flavus* CA14 (*kusA*^−^, *pyrG*^−^) strain. Transformants were verified using primers *AflZKS3*-iden-1 and *AflZKS3*-iden-2.

For the construction of the complementation strain, the native promoter, coding sequence, and terminator were amplified using primers *AflZKS3*-com-1 and *AflZKS3*-com-2, and ligated to the pPTRI plasmid after double digestion with *Hind*III and *Sma*I. After ligation, an ampicillin antibiotic was used to identify successfully constructed recombinant plasmids, and these were transferred to Δ*AflZKS3* protoplasts according to a previous study [55]. Pyrithiamin-resistant transformants were selected, and PCR was used for verification.

For construction of the *AflZKS3*-eGFP localization strain, linker, enhanced green fluorescent protein (eGFP), and TglaA (primers *AflZKS3*-eGFP-3, *AflZKS3*-eGFP-4) were connected to the *AFLA_026410* gene in sequence according to a previous study [54], and fused PCR products were purified and linked to the pPTRI plasmid after *Hind*III and *Sma*I double digestion. The successfully ligated plasmid was transferred into Δ*AflZKS3* protoplasts, and transformants were selected for PCR verification.

### 4.4. Localization Analysis of AflZKS3 in A. flavus

To assess the subcellular localization of *AflZKS3*, mycelium was grown for 12 h, collected, stained with 4′,6-diamidino-2-phenylindole (DAPI) according to a previous method [56], and analyzed using an Olympus FV1000 laser confocal microscope (Olympus, Beijing, China). DAPI and eGFP-labeled cells were sequentially imaged by dual-channel imaging.

### 4.5. Morphological and Physiological Analysis

In order to study the morphological effects of *AflZKS3* on *A. flavus*, 2 μL of spore suspension (10^6^ spores/mL) was inoculated on the surface of PDA medium, and after 5 days of incubation at 30 °C, colony size was observed, colony diameter was measured, the conidia number was calculated, conidia head morphology was assessed by stereoscopic microscopy, and the amount of toxin synthesis evaluated by thin layer chromatography (TLC). The number of conidia was used to calculate the conidia number of the whole plate, and then the area was obtained based on the colony diameter, and finally, the conidia number per cm^2^ was obtained. PDA medium was supplemented with 200 μg/mL CFW, 200 μg/mL Congo red (CR), 1 M NaCl, and 1.2 M sorbitol for stress testing. Additionally, scanning electron microscopy (SEM) was used to photograph the spore and conidia microstructure of the *A. flavus* control, Δ*AflZKS3* deletion, and Δ*AflZKS3*-Com strains, as previously described [57].

### 4.6. Extraction and Detection of AFs

*A. flavus* control, Δ*AflZKS3* deletion, and Δ*AflZKS3*-Com strains were inoculated in the middle of PDA medium and cultured in the dark for 5 days at 30 °C. Solid samples were collected, and AF was extracted from the culture using chloroform and separated via TLC. The developing solvent was chloroform: acetone (85:15). When the developing solvent migrated to 2/3 of the silica gel plate, the plate was removed to dry, and the fluorescence intensity of AFB_1_ was observed at a UV wavelength of 365 nm.

### 4.7. Evaluation of the Effect of AflZKS3 Deletion on the Growth of A. flavus Infecting Peanut and Corn

*A. flavus* was propagated on peanut and corn. Seeds were treated according to previous methods [54]. *A. flavus* control, Δ*AflZKS3* deletion, and Δ*AflZKS3*-com spore suspensions were inoculated with 10^6^ spores/ml and incubated in the dark for 5 days at 30 °C. A known amount of sterile water was added to the Petri dishes every day. Spore fluid was collected, and the number of conidia was counted by a hemocytometer according to a previous method [54]. Then, 20 mL of chloroform was added to detect the AFs as described above.

### 4.8. Transcriptome Analysis

Total RNA was extracted using a TRIzol kit (Thermo Fisher, Shanghai, China) according to the manufacturer’s instructions. RNA quality and RNA integrity were assessed, cDNA libraries were constructed, and RNA-seq analysis was performed by Guangzhou Gene Denovo Biotechnology (Guangzhou, China). Three biological replicates were set up for the RNA-seq analysis, and the accuracy and reliability of sample selection were analyzed with the Pearson correlation coefficient. DESeq was then used to analyze differences in FPKM value, and genes were considered differentially expressed when |log2 (fold change)| > 1 and *p*-value < 0.05 criteria were met. Gene Ontology (GO) terms and Kyoto Encyclopedia of Genes and Genomes (KEGG) pathways of DEGs were analyzed using GO and KEGG databases (http://www.genome.jp/kegg/, accessed on 12 October 2022).

### 4.9. Quantitative Real-Time PCR (qRT-PCR) Verification

To detect the expression of AF biosynthesis-related genes, spore suspensions (10^6^) were spread on a PDA plate and cultured at 30°C for 3 days. Total RNA was extracted using the above methods, and cDNA was synthesized by reverse transcription using the PrimeScrip™ RT reagent kit (Takara, Japan). qRT-PCR was performed using the Step One system (Applied Biosystems, Waltham, MA, USA), in which the β-actin housekeeping gene was used as the internal reference for normalization. Relative gene expression was calculated by 2^−ΔΔCt^. The qRT-PCR primers are listed in Table S1.

### 4.10. Data Analysis

Analysis of variance and least significant difference (LSD) tests were used for statistical analysis to determine the significance of differences between means, and *p* < 0.05 was considered statistically significant.

## Figures and Tables

**Table 1:** Representative DEG classification in Δ*AflZKS3* strain vs. *A. flavus* control.

Gene Category	Log_2_(fc)	Name	Description
**Growth**			
AFLA_046830	−2.37	*FLOT1*	flotillin domain protein
AFLA_089670	−1.38	*freB*	ferric reductase transmembrane component 4 precursor
AFLA_061400	−1.25	*aspC*	aminotransferase
AFLA_014230	−1.49	*psd2*	phosphatidylserine decarboxylase
AFLA_074470	−2.25	*vosA*	nuclear division Rft1 protein
AFLA_044800	−1.70	*con-6*	conidiation protein Con-6
AFLA_085140	−1.18	*cetA*	extracellular thaumatin domain protein
AFLA_058960	−1.03	*DIT2*	hypothetical protein AFLA_058960
AFLA_041620	−6.80	*AQY1*	aquaporin
AFLA_016100	−5.30	*betA*	glucose-methanol-choline (gmc) oxidoreductase
AFLA_122440	−1.40	*SAY1*	lipase/thioesterase family protein
**Cell wall**			
AFLA_006590	−1.19	*chiA*	class III chitinase ChiA1
AFLA_077910	−2.41	*agn1*	alpha-1,3-glucanase
AFLA_108860	−1.03	*gel2*	1,3-beta-glucanosyltransferase Gel2
AFLA_064920	1.96	*gel4*	1,3-beta-glucanosyltransferase gel4 precursor
AFLA_124160	−1.39	*glx3*	intracellular protease/amidase
AFLA_018750	−1.09	*gpi13*	phosphatidylinositol glycan
**Secondary metabolism**			
AFLA_038640	−1.12	*fasA*	fatty acid synthase alpha subunit
AFLA_139370	−1.31	*aflB*	aflB/fas-1/fatty acid synthase beta subunit
AFLA_093600	−2.77	*aflF*	oxidoreductase
AFLA_002920	−1.94	*aflQ*	flavonoid 3-hydroxylase
AFLA_064290	−4.26	*imqG*	O-methyltransferase
AFLA_059990	−3.93	*aclH*	O-methyltransferase
AFLA_101720	−2.06	*lnaC*	cytochrome P450
AFLA_097510	−8.69	*BOT4*	cytochrome P450 monooxygenase
AFLA_118990	−2.08	*gliA*	efflux pump antibiotic resistance protein
AFLA_006170	−1.43	*albA*	polyketide synthetase PksP
AFLA_060010	−4.70	*nscA*	PKS-like enzyme
AFLA_127090	−1.43	*pksCT*	polyketide synthase
AFLA_101700	−2.34	*lnaA*	NRPS-like enzyme
**Oxidative stress**			
AFLA_033420	−2.26	*sodB*	Mn superoxide dismutase MnSOD
AFLA_034380	−2.25	*cat1*	catalase
AFLA_124620	−9.14	*oxr1*	disulfide oxidoreductase
AFLA_117020	−3.24	*ssuD*	alkanesulfonate monooxygenase
AFLA_089810	−2.34	*dur3*	sodium/solute symporter
AFLA_075170	−2.26	*phoD*	alkaline phosphatase

## Data Availability

The raw transcriptome read data are available in the SRA database under accession number SUB12290388.

## Supplementary Materials

The following supporting information can be downloaded at: https://www.mdpi.com/article/10.3390/toxins14120883/s1, Figure S1: Construction and verification of Δ*AflZKS3*, Δ*AflZKS3*-Com, and *AflZKS3*-eGFP strains; Figure S2: Quality control of samples and overview of DEGs; Figure S3: Location of *AflZKS3*-eGFP in *A. flavus* in the presence of CFW, NaCl, and sorbitol; Table S1: Primers used in this study.
