# Supplementary material for: Putative C2H2 Transcription Factor AflZKS3 Regulates Aflatoxin and Pathogenicity in Aspergillus flavus
Source: Toxins (Basel). 2022 Dec 17;14(12):883. doi: 10.3390/toxins14120883 (PMC9786134; doi:10.3390/toxins14120883)
Supplement: Supplementary file 1 [file toxins-14-00883-s001.zip › toxins-2060713-supplementary.pdf]

toxins

MDPI

Supplementary Materials

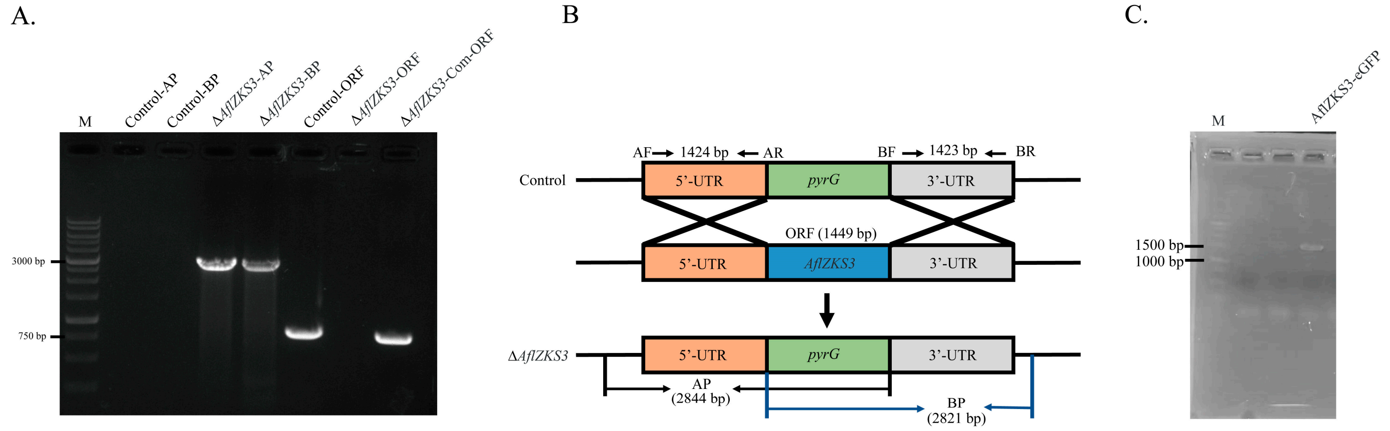

Figure S1. Construction and verification of Δ*AflZKS3*, Δ*AflZKS3*-Com and *AflZKS3*-eGFP strains. (A) Verification of Δ*AflZKS3* and Δ*AflZKS3*-Com strains. Control-AP and Δ*AflZKS3*-AP were amplified with primers *AflZKS3*-del-1 and pyrG-R. Control-BP and Δ*AflZKS3*-BP were amplified with primers pyrG-F and *AflZKS3*-del-4. AP represented a fragment including the upstream homologous arm of *AflZKS3* and pyrG, and BP represented a fragment including pyrG and the downstream homologous arm of *AflZKS3*. Control-ORF, Δ*AflZKS3*-ORF and Δ*AflZKS3*-Com-ORF were amplified with primers *AflZKS3*-iden-1 and *AflZKS3*-iden-2 localized with the ORF of *AflZKS3*; (B) Schematic diagram of Δ*AflZKS3* strain construction; (C) Verification of *AflZKS3*-eGFP strain. *AflZKS3*-eGFP were amplified with primers *AflZKS3*-iden-1 and *AflZKS3*-eGFP-4 to ensure the fusion protein *AflZKS3*-eGFP was constructed successfully.

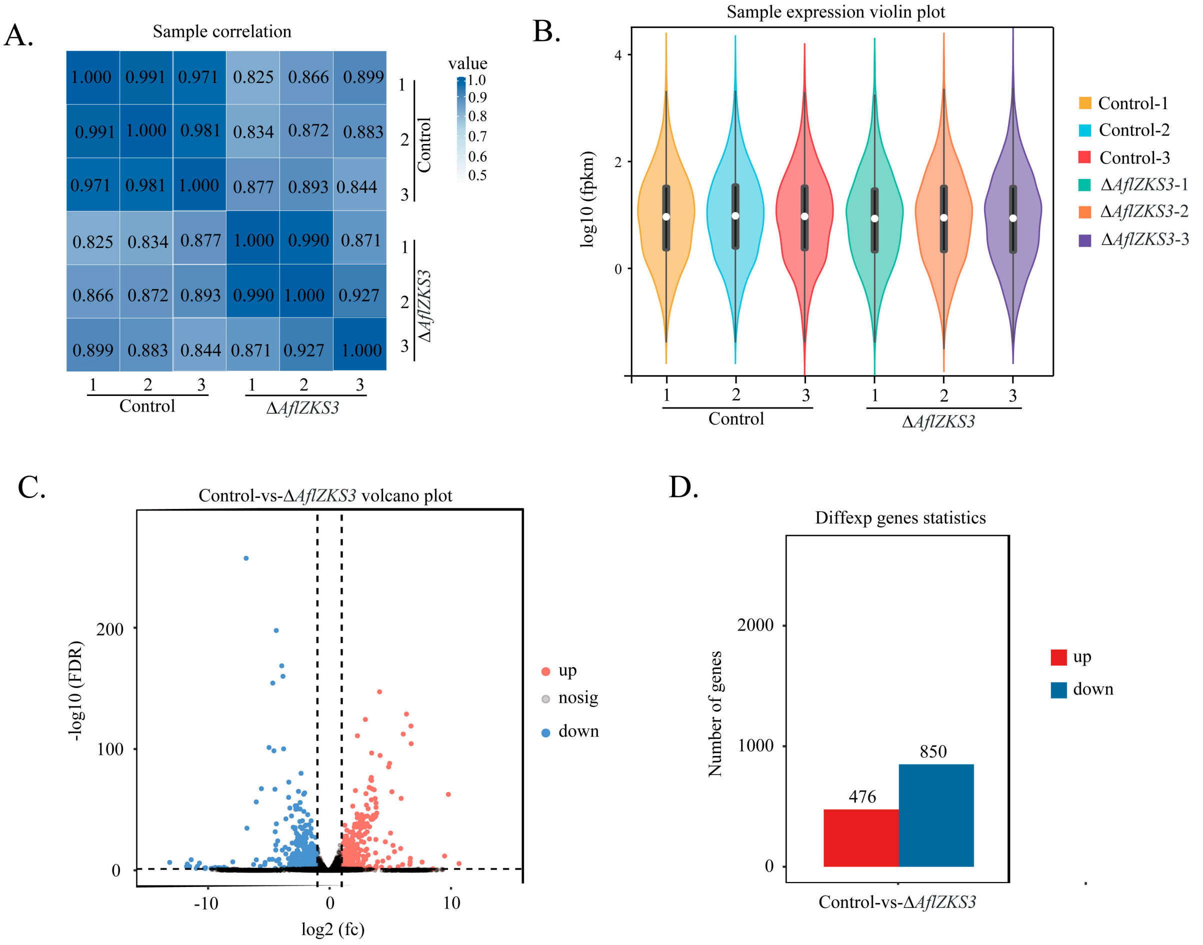

Figure S2. Quality control of samples and overview of DEGs. (**A**) Person correlation coefficient analysis; (**B**) Violin plot; (**C**) Volcano map of DEGs; (**D**) Number of DEGs.

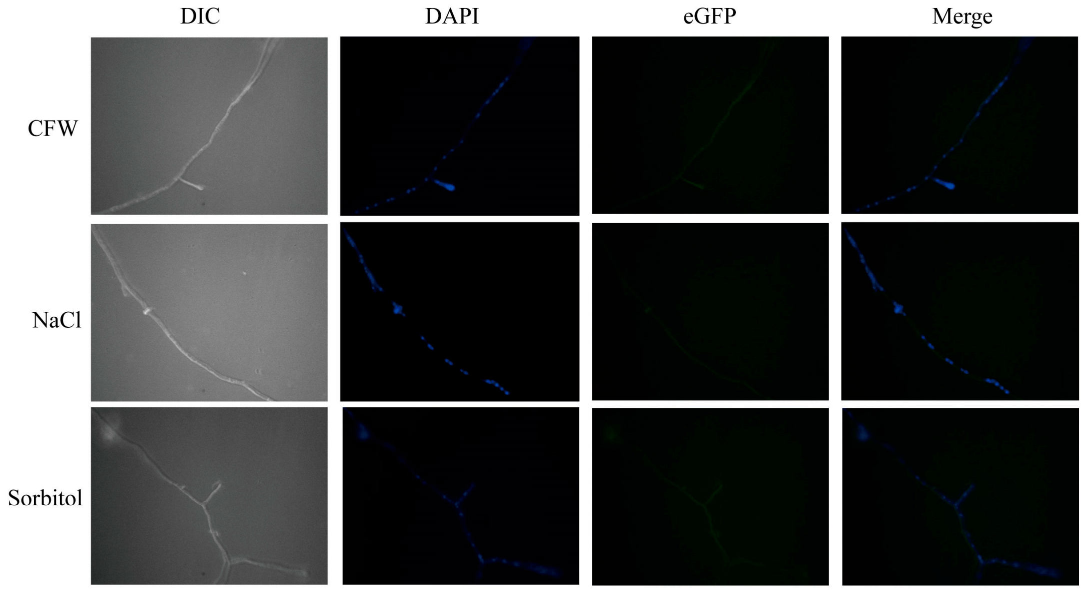

Figure S3. Location of *AflZKS3*-eGFP in *A. flavus* in the presence of CFW, NaCl and sorbitol.

Table S1. Primers used in this study.

Primer name	Sequence (5′-3′)	Application
AflZKS3-del-1	ctagaggatctactagtcatatggattTCATCTACTGTTTTCCACCCCCT	Δ*AflZKS3* strain construction
AflZKS3-del-2	ctgcggcgcgttctcgaggaagttgcAGGGACGGTAGTCAAGGACAAAT	
AflZKS3-del-3	atgtgtaacggtattgactaaaagggAGTCTCAATGATCGGCAGGACCTCT	
AflZKS3-del-4	tcgagctcggtacccggggatccgattCAGACTCAATAGCCGACAGCAAGAC	
AflZKS3-iden-1	CGTGTCGCTATACTTCATTTGG	
AflZKS3-iden-2	TCATCTCGTAGTTCTCCGGTTC	
pyrG-F	GCAACTTCCTCGAGAACGCGCCGCA	
pyrG-R	CCCTTTTAGTCAATACCGTTACACA	
AflZKS3-com-1	AAACAGCTATGACCATGATTACGCCAGCTTGCAGCTTCACACCTGATAATA	Δ*AflZKS3*-Com strain construction
AflZKS3-com-2	GGCCAGTGAATTCGAGCTCGGTACCCGTACGGCGGATATTAAATGGATGAG	
AflZKS3-eGFP-1	AAACAGCTATGACCATGATTACGCCATTTTCTATTTGACTTGAACTCCACG	*AflZKS3*-eGFP strain construction
AflZKS3-eGFP-2	AATTTTTGGCTTCATCTCGTAGTTC	
AflZKS3-eGFP-3	GAACTACGAGATGAAGCCAAAAATTggcgcaggcgccggcgcaggcgccggcgca	
AflZKS3-eGFP-4	GGCCAGTGAATTCGAGCTCGGTACCCCCGGAGATCCTGATCATCCGTGAGA	
aflB-F	GCCATTCACCAAGTCATACA	qRT-PCR
aflB-R	AACCTACGCTCAAATCCGAT	
aflF-F	CGCAAACTTCAGACAACCTA	
aflF-R	ATCCCTTTATCTGAGCACGA	
aflQ-F	GTCGCATATGCCCCGGTCGG	
aflQ-R	GGCAACCAGTCGGGTTCCGG	
FLOT1-F	GATGTTGCTGAGGCACGGAT	
FLOT1-R	CCAGTGTCCAAATCAGCAAG	
actin-F	ACGGTGTCGTCACAAACTGG	
actin-R	CGGTTGGACTTAGGGTTGATAG	
